# National Hepatitis C estimates: Incidence, prevalence, undiagnosed proportion and treatment, Canada, 2019

**DOI:** 10.14745/ccdr.v48i1112a07

**Published:** 2022-11-03

**Authors:** Nashira Popovic, Anson Williams, Simone Périnet, Laurence Campeau, Qiuying Yang, Fan Zhang, Ping Yan, Jordan Feld, Naveed Janjua, Marina Klein, Mel Krajden, William Wong, Joseph Cox

**Affiliations:** 1 Centre for Communicable Diseases and Infection Control, Public Health Agency of Canada, Ottawa, ON; 2 Toronto Centre for Liver Disease, Toronto General Hospital, University Health Network, Toronto, ON; 3 British Columbia Centre for Disease Control, Vancouver, BC; 4 Department of Medicine, McGill University Health Center, Montréal, QC; 5 School of Pharmacy, University of Waterloo, Kitchener, ON

**Keywords:** Hepatitis C, prevalence, incidence, epidemiology, Canada

## Abstract

**Background:**

Estimates of the number of hepatitis C virus (HCV) infections are important for monitoring efforts aimed at preventing disease transmission, especially following the introduction of a highly effective treatment. This report provides updated estimates of HCV incidence, prevalence, undiagnosed proportion and treatment in Canada.

**Methods:**

A combination of back calculation modelling and a modified version of the workbook method were used to estimate the incidence and prevalence of anti-HCV positive persons, the prevalence of chronic HCV infection and the undiagnosed proportion. The number of people treated for chronic HCV was estimated using administrative pharmaceutical data.

**Results:**

An estimated 9,470 new infections occurred in 2019, corresponding to an incidence rate of 25 per 100,000 population, a 7.7% decrease since 2015. The estimated prevalence of anti-HCV antibodies in the Canadian population was 1.03% (plausible range: 0.83%–1.38%), and the estimated prevalence of chronic HCV was 0.54% (plausible range: 0.40%–0.79%). The overall proportion of anti-HCV positive persons who were undiagnosed was estimated at 24% of all infections, with individuals born between 1945 and 1975 being the priority population the most likely to be undiagnosed. An estimated 74,500 people with chronic HCV have been treated since the introduction of direct-acting antivirals in 2014.

**Conclusion:**

Estimates of HCV incidence and prevalence are key metrics to guide interventions and resource allocation. While our estimates show that HCV incidence has decreased in Canada in recent years and treatment of chronic HCV has continued to increase, ongoing efforts are required to reduce the burden of HCV in Canada.

## Introduction

Globally, an estimated 58 million people have chronic hepatitis C virus (HCV) infection, with about 1.5 million new infections occurring per year ( ((1))). The number of people living with HCV has continued to increase, even though an effective cure exists ( ((2))). Canada has developed a pan-Canadian framework for action ( ((3))) as well as an accompanying Government of Canada five-year action plan ( ((4))) to help guide Canada’s efforts towards reducing the health impacts of sexually transmitted and blood-borne infections (STBBIs) in Canada by 2030.

The Global Health Sector Strategies on human immunodeficiency virus (HIV), viral hepatitis and sexually transmitted infections introduced targets for viral hepatitis control and elimination by 2030 ( ((2))). These include targets for the following: reduction of the annual number of new infections overall and among people who inject drugs; the reduction of the number of deaths from HCV; and an increase in the proportion of people living with HCV who have been diagnosed and cured. While the Government of Canada endorses these global targets, the first priority of the pan-Canadian STBBI action plan ( ((4))) is to develop domestic indicators and targets that will allow for the monitoring of Canada’s progress.

This report provides an update for 2019 on Canada’s estimates of HCV incidence, prevalence, proportion of undiagnosed and treated cases, which supports the Government of Canada’s commitment to monitor and report on progress towards hepatitis C elimination.

## Methods

A combination of back-calculation statistical modelling ( ((5))) and a modified version of the workbook method ( ((6))) were used to estimate new anti-HCV seropositivity (incidence), prevalence of anti-HCV positive persons (i.e. persons who have ever been infected with HCV), the prevalence of ribonucleic acid (RNA)-positive persons (i.e., persons with active infection, as a proxy for chronic HCV infection) and the undiagnosed/unaware proportion of the population. This methodology was developed and refined through a series of consultations that took place between 2019 and 2022. Experts from a variety of backgrounds were consulted, including hepatologists, research epidemiologists, laboratory specialists and mathematical modellers.

### Back calculation modelling

Back calculation is a widely used computational method to infer disease infections—which are not observable—from consequential results such as reported diagnostic cases. The method was initially designed to estimate the HIV/acquired immunodeficiency syndrome incidence ( ((5))) and was later adopted to estimate Canadian HCV incidence and prevalence for 2011 ( ((7))). Following the same approach, back calculation modelling was conducted using HCV routine surveillance data from the Canadian Notifiable Diseases Surveillance System, extracted on October 22, 2021. All reported cases (acute, chronic and unspecified) from 1991 to 2019 from five large Canadian provinces (British Columbia, Alberta, Saskatchewan, Ontario and Québec) were used. These provinces, which represented 90% of the Canadian population in 2019 ( ((8))), are the only ones who provide record-level HCV surveillance data. Modelled results were then extrapolated to the entire country. More information on the modelling can be found in **Appendix A**.

### Modified workbook method

The workbook method is an established approach previously used to produce estimate of HIV prevalence in low level and concentrated HIV epidemics ( ((6))). A modified version of this method was used to estimate the number of anti-HCV positive persons as well as their diagnosis status, and the number of HCV RNA-positive persons in Canada. We divided the Canadian population into subgroups that are known to be at higher risk of infection, and synthesized published and unpublished data to estimate prevalence within each subgroup. Each anti-HCV seroprevalence measure was classified as an ”underestimate”, ”overestimate” or ”appropriate estimate” based on a review of the methodology of each study. The under and over-estimates were used as plausible ranges of the appropriate estimates.

Estimates of the population size of each subgroup in Canada were based on data from Statistics Canada ( ((8–10))), as well as unpublished data obtained through unpublished datas, as detailed in the systematic review section. Point estimates of HCV prevalence were produced along with their upper and lower bounds by multiplying the HCV prevalence by the corresponding population size estimate.

The workbook subgroup populations were based on the following priority populations, as outlined in the *Blueprint to inform hepatitis C elimination efforts in Canada* ( ((11))):

• People who inject drugs (PWID)

• Adults in the 1945–1975 birth cohort

• Immigrant populations

• Indigenous peoples (First Nations, Inuit and Métis)

• Gay, bisexual and other men who have sex with men (gbMSM)

• People who are incarcerated (PWAI) in federal and provincial prisons

Due to the extensive overlap between these priority populations, they were not considered to be mutually exclusive.

### Systematic review

A health librarian at the Public Health Agency of Canada conducted a series of literature searches to obtain data on 1) HCV incidence and prevalence in Canada from January 1, 2019, to October 1, 2021, and 2) the unaware/undiagnosed proportion of HCV infection in Canada from January 1, 2016, to October 1, 2021. The literature searches yielded an initial 1,187 records, with an additional 31 records found outside of the librarian search. Using the systematic review protocol for prevalence and incidence studies developed by Joanna Briggs Institute ( ((12))), two independent reviewers screened all studies for inclusion. Discrepancies between reviewers were resolved through discussion. A total of 43 records were included after the final review and considered for use in the workbook method. Details about this process can be found in **Appendix B**.

In addition to the sources identified through the systematic review, unpublished data were requested from organizations and researchers. These sources included Canadian Blood Services (*unpublished data, Canadian Blood Services, 2022*) and Héma-Québec (*unpublished data, Héma-Québec, 2022*), Correctional Services Canada (*unpublished data, Correctional Services Canada, 2022*), Tracks bio-behavioural survey data (*unpublished data, Public Health Agency of Canada, Tracks bio-behavioural surveys, 2022*), and the Engage cohort study (*unpublished data, Engage cohort study, 2022*).

### Chronic hepatitis C prevalence and undiagnosed proportion estimates

**Figure 1:**
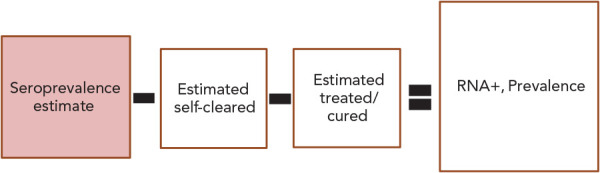
Equation used to estimate chronic hepatitis C prevalence, overall Canadian population

The overall seroprevalence estimate derived from the workbook method was used as the starting point to estimate the overall chronic hepatitis C prevalence in Canada (**Figure 1**). First, we subtracted the estimated number of individuals who had spontaneously cleared the virus, using a 30% clearance estimate based on a range of clearance proportions measured in Canadian studies ( ((13–17))). We then subtracted the estimated number of cured individuals, which was calculated using Canadian treatment estimates from the British Columbia Centre for Disease Control (2012–2016) (*unpublished data, British Columbia Centre for Disease Control, 2022*) and IQVIA (2017–2019) (*unpublished data, IQVIA, 2022*), using a cure rate of 48% for 2012–2014, and of 90% for 2015–2019. This calculation yielded a remaining number of HCV RNA-positive individuals in Canada, which was used as a proxy for chronic HCV infection.

**Figure 2:**
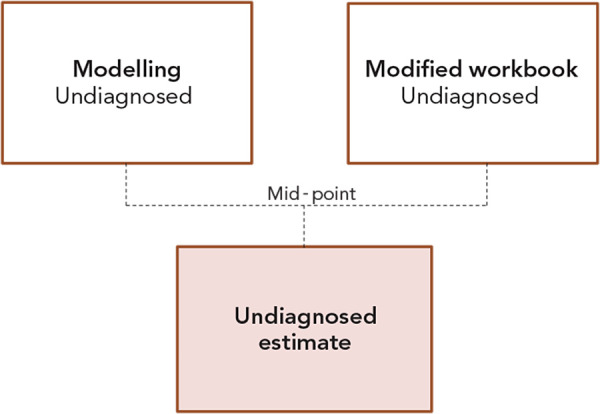
Equation used to estimate the undiagnosed proportion

Lastly, the estimate of the undiagnosed/unaware proportion of anti-HCV infection in Canada was determined by taking the midpoint between the back calculation modelling estimate and the modified workbook estimate (**Figure 2**). This approach was chosen to minimize the uncertainty that is inherent to estimates, which are partly based on assumptions due to the incompleteness of available data. Although uncertainty can never be completely eliminated, the true number likely lies between those two estimates.

## Results

### Hepatitis C virus incidence

**Figure 3:**
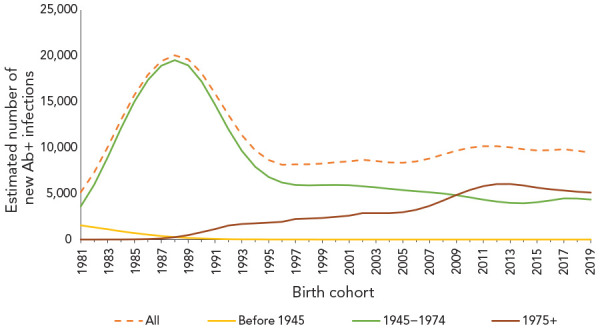
Estimated number of new hepatitis C infections by birth cohort, Canada, 1980–2019 Abbreviation: Ab+, antibody-positive

According to back calculation modelling, an estimated 9,470 new HCV antibody-positive infections occurred in 2019, corresponding to an annual incidence rate of 25 per 100,000 population. When modelled by birth cohort, the highest annual incidence was estimated among persons born after 1974 at 5,115 new infections, followed by persons born between 1945 and 1974 at 4,354 new infections. There were no new HCV infections estimated among persons born before 1945 (**Figure 3**).

### Hepatitis C virus treatment

**Figure 4:**
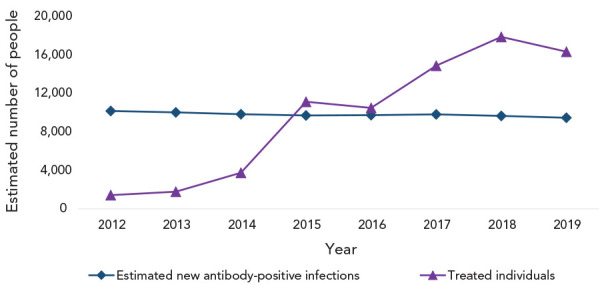
Estimated number of new hepatitis C infections and estimated number of people treated, Canada, 2012–2019

We estimate that since the introduction of direct-acting antivirals in Canada in 2014, approximately 74,500 people living with chronic HCV were treated, with 65.9% of those treatments having occurred between 2017 and 2019. **Figure 4** shows the yearly number of individuals treated, contrasted with the estimated number of new HCV infections.

### Hepatitis C virus prevalence

Using the modified workbook method, the estimated prevalence of anti-HCV in Canada in 2019 was 1.03% (plausible range: 0.83%–1.38%) or 387,000 (plausible range: 312,000–519,000) persons. Among priority populations, the highest prevalence of anti-HCV was among PWID (past 6–12 months) at 46.1% (plausible range: 28.0%–64.2%), followed by those with a lifetime history of infection drug use at 44.9% (plausible range: 25.6%–64.2%). Anti-HCV prevalence was also significantly higher among PWAI and Indigenous peoples than among the general population, at 10.7% (plausible range: 8.19%–13.2%) and 7.4% (plausible range: 3.49%–11.2%), respectively (**Table 1**).

**Table 1:** Estimated anti-hepatitis C antibodies positive prevalence by priority population, Canada, 2019

Population	Population size	Anti-HCV positive prevalence (%)			Number of anti-HCV positive persons			References
		Point estimate	Lower bound	Upper bound	Point estimate	Lower bound	Upper bound	
General population	37,601,230	1.03%	0.83%	1.38%	387,000	312,000	519,000	( ((8,18–21))) *Unpublished datas, Canadian Blood Services, 2022* *Unpublished data, Héma-Québec, 2022*
PWID—Current (PWID in the past 6–12 months)	133,651	46.1%	28.0%	64.2%	61,600	37,400	85,800	( ((18,22,23))) *Unpublished data, Williams A. Sorge J., 2022*
PWID—History (People who have a lifetime history of injection drug use)	389,574	44.9%	25.6%	64.2%	175,000	99,800	250,000	( ((18))) *Unpublished data, Williams A. Sorge J., 2022*
Adults in the 1945–1975 birth cohort	13,975,919	1.74%	1.27%	2.20%	242,000	177,000	307,000	( ((8,18–20,24–27)))
Immigrant population	11,778,177	1.51%	0.70%	2.32%	178,000	82,500	273,000	( ((18,20,28)))
Indigenous peoples (First Nations, Inuit, Métis)	1,826,356	7.35%	3.49%	11.2%	134,000	63,700	205,000	( ((18,29)))
gbMSM	640,785	3.70%	1.70%	5.10%	23,400	10,900	32,700	( ((30–32))) *Unpublished data, Engage cohort study, 2022*
People who are incarcerated—Federal and provincial	37,932	10.7%	8.19%	13.2%	4,050	3,110	5,000	( ((9,33–35))) *Unpublished data, Correctional Services Canada, 2022*

Abbreviations: gbMSM, gay, bisexual and other men who have sex with men; HCV, hepatitis C virus; PWID, people who have used injection drugs

Of the estimated number of persons ever infected with HCV (anti-HCV positive), an adjustment of 30% or 116,188 persons was made to account for individuals who spontaneously cleared HCV infection. A second adjustment of 67,018 persons was made to account for individuals who were cured of HCV infection through treatment. After adjusting for HCV clearance and treatment, the estimate of chronic HCV prevalence was 0.54% (plausible range: 0.40%–0.79%) or 204,000 persons (plausible range: 151,000–296,000).

Among priority populations, the highest prevalence rate of chronic HCV infection was among current PWID at 36.9% (plausible range: 12.6%–55.1%). The lowest prevalence rate among priority populations was found among adults in the 1945–1975 birth cohort at 0.9% (plausible range: 0.4%–1.3%) (**Table 2**).

**Table 2:** Estimated chronic hepatitis C infection prevalence by priority population, Canada, 2019

Population	Population size	Chronic hepatitis C prevalence (%)			Number of persons living with chronic hepatitis C			References
		Point estimate	Lower bound	Upper bound	Point estimate	Lower bound	Upper bound	
PWID—Current (PWID in the past 6–12 months)	133,651	36.9%	12.6%	55.1%	49,300	16,800	73,600	( ((18,22))) *Unpublished data, Tracks bio-behavioural surveys, Public Health Agency of Canada, 2022* *Unpublished data, Williams A. Sorge J., 2022*
PWID—History (People who have a lifetime history of injection drug use)	389,574	29.6%	22.3%	36.9%	115,000	87,000	144,000	( ((18,22,24)))
Adults in the 1945–1975 birth cohort	13,975,919	0.87%	0.44%	1.30%	122,000	61,500	182,000	( ((8,18,20)))
Immigrant population	11,778,177	Insufficient data						N/A
Indigenous peoples (First Nations, Inuit, Métis)	1,826,356	3.5%	2.0%	5.0%	63,900	36,500	91,300	( ((18,29,36)))
gbMSM	640,785	1.1%	0.4%	1.7%	7,050	2,560	10,900	( ((32))) *Unpublished data, Engage cohort study, 2022*
People who are incarcerated—Federal and provincial	37,932	3.7%	2.3%	5.1%	1,400	870	1,940	( ((35))) *Unpublished data, Correctional Services Canada, 2022*

Abbreviations: gbMSM, gay, bisexual and other men who have sex with men; N/A, not applicable; PWID, people who have used injection drugs

### Undiagnosed proportion

The overall proportion of anti-HCV positive persons in Canada who were undiagnosed or unaware of their HCV status was estimated at 24% or 79,500 persons (data not shown). This was calculated by taking the midpoint between the modelling estimate (n=60,200, 19.2%) and the modified workbook estimate (n=98,800, 25.5%). Among priority populations, the highest proportion of undiagnosed/unaware HCV infection was estimated among adults in the 1945–1975 birth cohort at 34.4% (plausible range: 18.8%–50.0%), followed by 22% among current PWID (plausible range: 18.5%–25.4%) and 22% among PWAI (plausible range: 12.3%–31.6%). The lowest proportion of undiagnosed/unaware was among the gbMSM population at 8.8% (plausible range: 6.7%–22.2%) (**Table 3**). The proportion of undiagnosed individuals could not be measured for people with a lifetime history of injection drug use, Indigenous peoples, and immigrant populations due to insufficient data.

**Table 3:** Estimated number and proportion of people unaware of their hepatitis C virus antibody-positive status by priority population, Canada, 2019

Population	Anti-HCV positive estimate	Undiagnosed/unaware (%)			Number of anti-HCV positive persons who were unaware/undiagnosed			References
		Point estimate	Lower bound	Upper bound	Point estimate	Lower bound	Upper bound	
PWID—Current (PWID in the past 6–12 months)	61,600	22.0%	18.5%	25.4%	12,400	10,500	14,300	Unpublished data, Tracks bio-behavioural surveys, Public Health Agency of Canada, 2022
PWID—History (People who have a lifetime history of injection drug use)	175,000	Insufficient data						N/A
Adults in the 1945–1975 birth cohort	242,000	34.4%	18.8%	50.0%	83,400	45,600	121,000	( ((27,37–40)))
Immigrant population	178,000	Insufficient data						N/A
Indigenous peoples (First Nations, Inuit, Métis)	134,000	Insufficient data						N/A
gbMSM	23,400	8.8%	6.7%	22.2%	2,060	1,570	5,200	Unpublished data, Engage cohort study, 2022
People who are incarcerated—Federal and provincial	4,050	22.0%	12.3%	31.6%	890	499	1,280	( ((34))) Unpublished data, Correctional Services Canada, 2022

Abbreviations: gbMSM, gay, bisexual and other men who have sex with men; HCV, hepatitis C virus; N/A, not applicable; PWID, people who have used injection drugs

## Discussion

The national hepatitis C estimates for 2019 provided updated insights into the hepatitis trends in Canada. These estimates will be used to support the pan-Canada five-year action plan on STBBI, with the goal of reducing the health impacts of STBBI in Canada by 2030. Based on our modelling, an estimated 9,470 new hepatitis C infections (25 per 100,000 population) occurred in 2019 in Canada, which corresponds to a reduction of 7.7% in incidence compared to 2015 (Figure 4). However, this reduction rate is insufficient to meet the 90% reduction in new chronic infections outlined in the World Health Organization 2030 elimination goals, thus confirming the need for continued efforts to curb HCV transmission and improve access to treatment for all HCV-infected individuals. We estimated that in 2019, approximately 1% of the Canadian population, or roughly 387,000 persons, were anti-HCV positive, meaning they were infected by the virus at some point in time (i.e., past or current infection). Of these individuals, an estimated 76% were diagnosed as anti-HCV-positive, leaving an estimated 24% who were unaware of their anti-HCV positive status. While this figure is encouraging, more progress needs to be made to reach the goal of 90% of people living with HCV being diagnosed by 2030. Of the different priority groups, baby boomers (e.g., adults born between 1945 and 1975) were the most likely to be undiagnosed.

Additionally, an estimated 204,000 persons, or approximately half of those who were estimated to be anti-HCV positive, were HCV RNA-positive in 2019, suggesting an active infection. Direct-acting antivirals are a cornerstone in treatment to reduce the risk of complications among those individuals and avert further transmission. Since this highly effective treatment was introduced in Canada in 2014, an estimated 74,500 people with chronic hepatitis C were treated. Encouragingly, our data also shows that between 2017 and 2019, the yearly number of treated individuals largely exceeded the number of new infections. As suggested elsewhere ( ((41))), maintaining high treatment uptake in the upcoming years will be essential to achieve HCV elimination in Canada by 2030.

Although our 2019 estimates confirmed that the burden of hepatitis C on the overall population is relatively low, certain populations and communities are disproportionately impacted. This is especially true for people who use injection drugs, who may face concomitant social, financial and health challenges and, therefore, require a more comprehensive approach to prevention, diagnosis and treatment. Other priority populations, including people who are incarcerated, Indigenous peoples and gbMSM, are also disproportionately affected. Targeted approaches, such as peer-supported and culturally competent outreach interventions, could be considered to reduce the burden of HCV among these groups.

## Strengths and limitations

Key strengths of our approach include the use of Canadian Notifiable Diseases Surveillance System data, a comprehensive database that encompasses all laboratory-confirmed cases of HCV in Canada. The combination of back-calculation and workbook methods also provides an opportunity to improve the overall estimates and increase accuracy. Our modified workbook approach allowed us to produce the first national HCV estimates specific to the priority populations based on the *Blueprint to inform hepatitis C elimination efforts in Canada* ( ((11))), thus making these data more actionable for policy-makers and service providers working with these groups.

Our analysis also has several limitations. First, estimates of HCV incidence were based on data on all reported cases (acute and chronic); therefore, the estimated incidence represents all individuals who developed anti-HCV antibodies. Separate estimates for the undiagnosed proportion among persons with acute and chronic infections could not be produced. Second, data by priority population were not available through routine national surveillance; therefore, national incidence estimates by priority population were not produced. As a result of these limitations, reporting on a full set of indicators against global targets was not possible at this time. Third, since individuals may identify as being members of more than one priority population, these categories are not mutually exclusive. However, unlike the workbook method used in previous national estimates, the modified workbook method does not use addition or subtraction between priority groups to yield an overall estimate for the general population. Instead, representative data for the general Canadian population were collected and a prevalence estimate was calculated independently of the other priority populations. Fourth, it was not possible to distinguish reinfections from initial infections; therefore, it is possible that individuals infected twice within the same year were counted twice in the yearly incidence estimates. Finally, treatment estimates were based on administrative pharmaceutical records of HCV treatment initiation; therefore, individuals who received HCV treatment through clinical trials or compassionate access may not be captured.

## Conclusion

Estimates of HCV incidence and prevalence can be used to guide health interventions and resource allocation to link chronically infected persons to screening, care, treatment and ultimately cure. While our estimates show that overall HCV incidence has been decreasing in Canada since 2010, continued efforts are required to eliminate chronic HCV as a public health threat by 2030. Significant progress towards HCV elimination will require targeted interventions to prevent new infections, especially among priority populations, innovative testing approaches to find undiagnosed persons and strategies to ensure linkage to care and prompt treatment. The Public Health Agency of Canada will continue to work closely with provinces and territories and other partners to enhance methods and data sources to improve the ability to measure and assess progress against elimination targets.

## Appendix

Appendix A: Methodology for back calculation modelling

Appendix B: Literature search

### Appendix A: Methodology for back calculation modelling

We use the same back calculation modelling approach as in the previous work for Canadian hepatitis C virus (HCV) estimation ( ((7))). In the back calculation modelling method, the time from HCV infection to diagnosis is considered a random variable that follows a certain probabilistic distribution. Once the transition probabilities *P* are known, the back calculation method calculates the expected number of infections *I* (as the estimated HCV incidence) through minimizing the gap between the reported HCV cases and the expected diagnosed HCV cases which is *P* x *I*. In the computation process, other modelling outcomes, such as expected HCV infections diagnosed in the same year, the expected HCV-related mortality and not-yet diagnosed HCV cases, are also produced.

The probabilities *P* are not known in advance, however, and it is assumed to follow a commonly used family of distribution called log-logistic distribution with a shape parameter and a scale parameter. As in the previous work ( ((7))), these parameters are determined from a wide range through iteratively searching the optimal fitting of the reported diagnosed cases, and HCV-related mortality data. In addition, the reported acute cases are also used to further calibrate the parameters by minimizing the gap between the acute cases and the expected HCV infections diagnosed in the same year. The calibration uses the standard BFGS-method available in R.

Using this method, HCV incidence was estimated by five-year birth cohort plus an extra open cohort (born after the year 2000). Five-year birth cohorts were then grouped into larger birth cohorts: before 1945, 1945–1974, and after 1975.

### Appendix B: Literature search

A health librarian at the Public Health Agency of Canada conducted a series of literature reviews based on the specific objectives of obtaining data on ( ((1))) HCV incidence and prevalence in Canada from January 1, 2019, to October 1, 2021, and ( ((2))) the unaware/undiagnosed proportion of HCV infection in Canada from January 1, 2016, to October 1, 2021. The following databases were searched by the Health Librarian for relevant publications: Ovid MEDLINE(R) ALL, Embase and Scopus. Additional grey literature searches were conducted using the Google search engine. In total, both literature searches yielded an initial 1,187 records, with an additional 31 records found outside of the librarian search.

Using the systematic review protocol for prevalence and incidence studies developed by Joanna Briggs Institute (JBI), two independent reviewers screened all studies for inclusion. For the initial screening, reviewers independently read either the abstract or the full text and made assessments based on the following inclusion criteria:

• Condition: HCV infection (past [seroprevalence] or present [active or chronic])

• Outcome:

o Literature search 1: Reporting data on proportion with HCV infection, prevalence or incidence

o Literature search 2: Reporting data on awareness of HCV infection, and/or undiagnosed proportion of people with HCV

• Context: In Canada

• Population: All populations used for the workbook method

After the initial screening, 66 records were included for final assessment. In the final assessment, both reviewers independently read and evaluated each paper using the *JBI Critical Appraisal Checklist for Studies Reporting Prevalence Data* to determine whether the paper should be included. Data from cross-sectional and cohort studies with a testing component were prioritized, but studies using administrative data and modelling studies were also included, when appropriate. Discrepancies between reviewers were resolved through discussion. After the final review, 43 records were included and considered for use in the workbook method (Tables 1, 2 and 3). Due to a limited number of records on the incidence and prevalence of HCV among gay, bisexual and other men who have sex with men (gbMSM) and baby boomers, a subsequent literature review with an extended date range of January 1, 2016, to December 31, 2018, was conducted for those two subgroups. This allowed us to find an additional seven records for inclusion in the workbook method.
